# Metabolic syndrome increases senescence-associated micro-RNAs in extracellular vesicles derived from swine and human mesenchymal stem/stromal cells

**DOI:** 10.1186/s12964-020-00624-8

**Published:** 2020-08-12

**Authors:** Yongxin Li, Yu Meng, Xiangyang Zhu, Ishran M. Saadiq, Kyra L. Jordan, Alfonso Eirin, Lilach O. Lerman

**Affiliations:** 1 Division of Nephrology and Hypertension, Mayo Clinic, 200 First Street SW, Rochester, MN 55905 USA; 2 Dapartment of Vascular Surgery, The Affiliated Hospital of Qingdao University, Qingdao, 266000 People’s Republic of China; 3 Department of Nephrology, The First Hospital Affiliated to Jinan University, Guangzhou, 510630 People’s Republic of China

**Keywords:** Metabolic syndrome, MSC, EV, RNA-sequencing, Senescence

## Abstract

**Background:**

The metabolic syndrome (MetS) is a combination of cardiovascular risk-factors, including obesity, hypertension, hyperglycemia, and insulin resistance. MetS may induce senescence in mesenchymal stem/stromal cells (MSC) and impact their micro-RNA (miRNA) content. We hypothesized that MetS also alters senescence-associated (SA) miRNAs in MSC-derived extracellular vesicles (EVs), and interferes with their function.

**Methods:**

EVs were collected from abdominal adipose tissue-derived MSCs from pigs with diet-induced MetS or Lean controls (*n* = 6 each), and from patients with MetS (*n* = 4) or age-matched Lean controls (*n* = 5). MiRNA sequencing was performed to identify dysregulated miRNAs in these EVs, and gene ontology to analyze their SA-genes targeted by dysregulated miRNAs. To test for EV function, MetS and Lean pig-EVs were co-incubated with renal tubular cells in-vitro or injected into pigs with renovascular disease (RVD, *n* = 6 each) in-vivo. SA-b-Galactosidase and trichrome staining evaluated cellular senescence and fibrosis, respectively.

**Results:**

Both humans and pigs with MetS showed obesity, hypertension, and hyperglycemia/insulin resistance. In MetS pigs, several upregulated and downregulated miRNAs targeted 5768 genes in MSC-EVs, 68 of which were SA. In MetS patients, downregulated and upregulated miRNAs targeted 131 SA-genes, 57 of which overlapped with pig-EVs miRNA targets. In-vitro, MetS-MSC-EVs induced greater senescence in renal tubular cells than Lean-MSC-EVs. In-vivo, Lean-MSC-EVs attenuated renal senescence, fibrosis, and dysfunction more effectively than MetS-MSC-EVs.

**Conclusions:**

MetS upregulates SA-miRNAs in swine MSC-EVs, which is conserved in human subjects, and attenuates their ability to blunt cellular senescence and repair injured target organs. These alterations need to be considered when designing therapeutic regenerative approaches.

**Video abstract**

**Graphical abstract:**

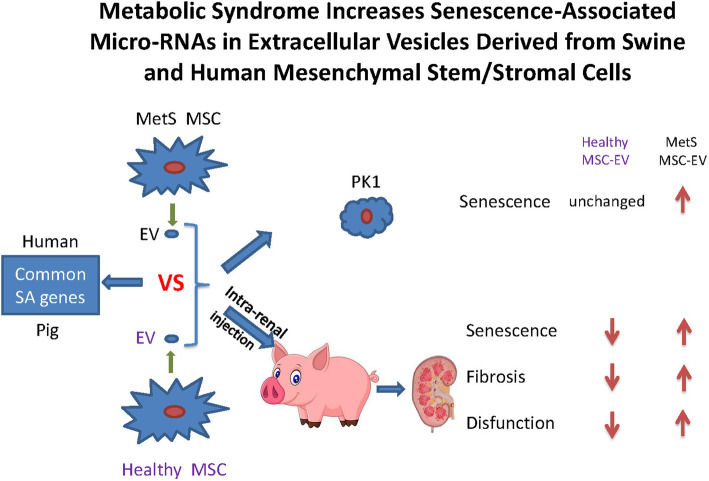

## Background

Mesenchymal stem cells (MSCs) are multipotent cells with high proliferative, self-renewal, multi-lineage differentiation, and regenerative potential [1]. Emerging evidence suggests that MSCs isolated from fat and other tissue sources feature potent immunomodulatory and proangiogenic properties [2, 3], and thus serve important reparative functions.

Cellular senescence is an important cell fate, which entails apoptosis-resistance, stable replicative arrest, acquisition of a pro-inflammatory, tissue-destructive senescence-associated (SA) secretory phenotype (SASP), and shifted metabolism [4]. During this process, fatty acid oxidation is decreased, but protein synthesis and generation of reactive oxygen species increase [5]. SASP is linked to a variety of chronic diseases, geriatric syndromes, and metabolic dysregulation, including metabolic syndrome (MetS) and atherosclerosis.

Characterized by dyslipidemia, obesity, insulin resistance, inflammation, as well as hypertension, MetS affects a large number of people worldwide [6]. The presence of MetS has been associated with atherosclerosis renovascular disease (RVD) and chronic kidney disease [7]. Accumulating evidence has shown that the proliferation capacity of MSCs is inversely correlated with cellular senescence and apoptosis in MetS patients [8]. We have also previously shown that MetS impairs the functionality of MSCs, increases their cellular senescence [9], and alters their genetic and protein content [10–12].

Previous studies [13, 14] showed that MSCs bestow their reparative effects by releasing extracellular vesicles (EVs), including micro-vesicles and exosomes. EVs can alter transcription profiles in recipient cells, and modulate tissue metabolism and cellular pathways. Our group recently demonstrated that EVs released by pig MSCs are selectively packed with micro-RNAS (miRNAs), mRNAs, and proteins, which have the capacity to modify selective pathways in recipient cells [10, 12, 15, 16]. miRNAs are small non-coding RNAs that regulate gene expression post-transcriptionally. We have shown that MetS modulates MSC expression of senescence-associated (SA)-miRNAs [12], but whether this alteration extends to their paracrine EVs, or interferes with their ability to suppress SA mechanisms in target cells, remains known.

The current study was therefore designed to test the hypothesis that MetS upregulates SA-miRNAs in adipose tissue MSCs-derived EVs in pigs, and impairs their repair capacity. Furthermore, we hypothesized that this adverse dysregulation would also be conserved in MSCs obtained from human subjects with MetS. For this purpose, we comprehensively evaluated the MSC-derived EV miRNA expression in both human subjects and pigs with MetS using high-throughput RNA sequencing, and injected pig EVs into injured kidneys of a pig model with RVD. A chief finding of our study is that miRNA spectrum in MSC-derived EVs in MetS might be involved in senescence-regulation. Importantly, we found that MetS stifles the ability of MSC-derived EVs to ameliorate senescence and fibrosis in RVD pig kidneys, and increases their propensity to induce senescence in target cells in vitro. These observations may have important implications for the reparative potency of endogenous EVs and for the utility of autologous exogenous EVs in subjects with MetS.

## Materials and methods

### Animals and protocol

Animal studies were approved by the Institutional Animal Care and Use Committee. For EV characterization, 12 three-months-old female domestic pigs (Manthei Hog Farm, Elk River, MN) were observed for 16 weeks in two groups. Control pigs (Lean) were fed a standard chow (13% protein, 2% fat, 6% fiber, Purina Animal Nutrition LLC, MN), and MetS pigs a metabolic-atherosclerotic diet (5B4L, protein 16.1%, ether extract fat 43.0%, and carbohydrates 40.8%, Purina Test Diet, Richmond, IN) (*n* = 6 each) [17]. After completion of diet, systemic parameters including body weight, blood pressure (measured with an intra-arterial catheter), and venous levels of cholesterol, triglycerides, fasting glucose, and insulin were measured by standard procedures [18]. MSCs and EVs were then collected.

### Patient population

Healthy subjects and MetS patients (*n* = 5 in each group) were recruited in the First Hospital Affiliated to Jinan University (Guangdong, China). The study followed the Declaration of Helsinki. Both the project and molecular testing were approved by the Institutional Research Ethics Committee, and written informed consent obtained from all subjects. All study participants were reviewed for medical history.

Inclusion criteria for MetS patients included age > 18 years old and MetS diagnosis, based on the criteria of the International Diabetes Federation. The diagnosis of the MetS was based on obesity (excessive waist circumference or BMI > 30 kg/m2) and two or more of the following factors: abnormal lipids metabolism (HDL cholesterol < 40 mg/dL in males and < 50 mg/dL in females, triglycerides ≥150 mg/dL), systolic blood pressure ≥ 130 mmHg or diastolic blood pressure ≥ 85 mmHg; fasting plasma glucose concentration ≥ 100 mg/dl or previously diagnosed hypertension or type-2 diabetes. Exclusion criteria for MetS patients included drug abuse, heavy smoking, cancer, severe heart valves diseases, and any kind of severe systemic diseases.

Inclusion criteria for healthy controls included age > 18 years, overall healthy individuals, who had abdominal fat harvested for cosmetic reasons. Exclusion criteria for healthy controls included drug abuse and heavy smoking.

Blood samples were collected prior to surgery for assessment of metabolic and renal function in the clinical laboratories of the First Hospital Affiliated to Jinan University. Estimated glomerular filtration rate (eGFR) was calculated by the MDRD eGFR Equation [19].

### MSCs and EV harvesting

Abdominal subcutaneous adipose tissue collection, MSC isolation and culture, and EV collection were performed as previously described [17].

Pigs were euthanized with a lethal intravenous dose of 100 mg/kg of sodium pentobarbital (Fatal Plus, Vortech Pharmaceuticals, Dearborn, MI, USA) and subcutaneous abdominal adipose (5–10 g) tissue immediately collected. Fat was digested in collagenase-H, filtered with 0.2-mm syringe filter, and cultured in advanced minimal essential medium (GIBCO/Invitrogen, Grand Island, NY, USA) supplemented with 5% platelet lysate for about 3 weeks. EVs were isolated from supernatants of passage 3 MSCs (at least 10^7^ cells) by ultracentrifugation, as previously described [10, 11]. Then the samples were centrifuged (2000 g for 20 min), and the supernatant collected and subsequently centrifuged (100,000 g for 1 h) at 4 °C. EVs were collected, suspended (wash-buffer medium 199) and centrifuged again (100,000 g for 1 h).

MSCs were previously characterized by the expression of CD44, CD90, and CD105, and lack of expression of CD45, CD34, CD14, using immunofluorescent staining and flow-cytometry [17]. Isolated EVs were then characterized based on the expression of EV (CD9, CD29, CD63) and MSC (CD73, CD105) markers by Western blot [17]. In human subjects, adipose tissue (5–10 g) was collected during bariatric (MetS patients) or cosmetic (Lean) surgeries. The samples were placed on ice and processed for MSC harvesting, expansion, and EV collection using identical methods to those employed in pig studies.

### MiRNA sequencing and data analysis

Human EV total RNA libraries were prepared with QIAseq Stranded Total RNA Kit. MiRNA sequencing libraries prepared with QIAseq miRNA Library Kit were sequenced using an Illumina NGS system (MiSeq Personal Sequencer, NextSequence500, HiSeq 1000, HiSeq 1500, HiSeq 2000, HiSeq 2500, and GaIIx). The data were analyzed with CLC (Biomedical) Genomics Workbench.

Sequencing RNA libraries of pig EVs were prepared according to the manufacturer’s protocol (TruSeq RNA Sample Prep Kit v2, Illumina). The pig EV data were analyzed using the CAP-miRSeq-v1.1 workflow [20]. The workflow starts with unaligned FASTQs, which generates aligned BAMs, and then excel sheets containing both raw and normalized known mature miRNA expression counts. The R-based tool from Bioconductor, edgeR2.6.2 was used to perform the differential expression analysis to identify miRNAs enriched in MetS-EVs compared to Lean-EVs (fold-change > 2.0 and *p* < 0.05). ComiR and TargetScan 7.1 were used to predict target genes of significantly upregulated and downregulated miRNAs. Subsequent functional annotation-clustering analysis utilized the DAVID 6.7 database.

### Validation of miRNA expression

To validate expression of representative miRNAs in pig EVs, the expression of miR-199a-5p, miR-132 and miR-99b was also measured by quantitative polymerase chain reaction (qPCR). Total RNA was isolated from MSC-derived EV samples [21], and probed with primers (TermoFisher Scientifc, Minneapolis, MN, USA; Catalog Numbers: miR-99b: 002196, miR-132: 000457, miR-199a-5p:000498).

### In vitro EV efficacy

For in vitro studies, pig renal tubular cells (PK1 cells, ATCC, Manassas) were cultured in Medium-199 (Gibco BRL, USA) containing 3% FBS15. EVs were incubated in 2 × 10^−6^M PKH26 for 5 min and culture media with 10% EV-depleted FBS (2 ml) added to stop the action. Then EVs were centrifuged (100,000 g for 1 h) and re-suspended in DPBS (5 ml). Pre-labeled EV-rich fractions (75 × 10^12/mL) were incubated with PK1 cells for 48 h, and tracked to confirm engraftment. Staining for SA-Spider-ß-Gal in PK1 cells was performed as previously described [22] following manufacturer’s protocol (Cat#SG04–01, Dojindo).

### In vivo EV efficacy

The pig RVD model and EV injection was performed as previously described [17, 23]. Twenty-four additional female domestic pigs were randomized into four groups: Lean, RVD, RVD + Lean-MSC-EVs and RVD + MetS-MSC-EVs (*n* = 6 each). Lean pigs were fed a standard chow, and RVD pigs a metabolic-atherosclerotic diet. Six weeks after initiation of diet, unilateral renal artery stenosis was induced in 18 RVD pigs by placing an irritant coil in the main renal artery [23] to achieve gradual arterial narrowing.

Six weeks after induction of RVD, EV-rich fractions from the Lean and MetS MSCs were labeled with the red fluorescence dye PKH26 (Sigma-Aldich, ST. Louis, MO). After incubating in 2 × 10^−6^MPKH26 for 5 min, EVs were added with culture media with 10% EV-depleted FBS (2 ml) to stop the action according to instructions. EVs were centrifuged (100,000 g for 1 h) and re-suspended in DPBS (5 ml). In two RVD groups, Lean or MetS EVs (100 μg or 10^11^) in 10 ml were then injected (each in 6 RVD pigs) through a catheter placed in the stenotic renal artery under fluoroscopic guidance. The other groups underwent renal angiography to determine the degree of stenosis, and all pigs were allowed to recover.

Four weeks after EV injection, blood pressure was measured using an intra-arterial catheter, the pigs were euthanized, and the kidneys harvested. The labeled EVs were tracked in frozen sections of RVD kidneys to confirm engraftment [11]. Staining for SA-Spider-ß-Gal was performed following manufacturer’s protocol (Cat#SG04–01, Dojindo), on 10 μm frozen sections counter-stained with eosin. Senescence (green) area in kidney was measured by using the Image-J software in 10 individual fields, calculated as percent of kidney area, and averaged for all fields.

Fibrosis was assessed using Masson’s trichrome staining with a modified IMEB stain kit (K7298, IMEB, San-Marcos, CA). Images were visualized with a Zeiss microscope. Fibrosis area (blue) in kidney was measured by MATLAB in 10 individual fields and averaged.

### Statistical analysis

Statistical analysis was performed using JMP 14.0 (SAS Institute, Cary, NC). Data were expressed as mean ± standard deviation. Comparisons among groups were performed using unpaired Student’s t-test and ANOVA. Nonparametric tests (Wilcoxon and Kruskal Wallis) was used when data did not follow a Gaussian distribution. Statistical significance was accepted if *p* ≤ 0.05.

## Results

### Systemic characteristics of Lean and Mets pigs

Table 1 shows the systemic characteristics of the Lean and MetS pigs. Compared with Lean, after 16 weeks of diet MetS pigs showed higher body weight, blood pressure, and cholesterol levels, including total cholesterol, LDL, and triglycerides. Furthermore, HOMA-IR score and fasting insulin were higher in MetS vs. Lean, although fasting glucose levels were unchanged. These findings indicate development of MetS.

**Table 1:** Systemic characteristics in experimental pig groups (*n* = 6 each) at 16 weeks

Parameter	Lean	MetS
Body weight (Kg)	71.4 ± 10.8	90.8 ± 1.8*
Mean blood pressure (mmHg)	95.1 ± 9.9	119.8 ± 3.9*
Total cholesterol (mg/dl)	80.8 ± 6.4	398.1 ± 60.0*
LDL cholesterol (mg/dl)	33.1 ± 5.2	378.1 ± 137.6*
Triglycerides (mg/dl)	7.7 ± 1.2	14.6 ± 1.5*
Fasting glucose (mg/dl)	127.1 ± 14.2	118.8 ± 18.0
Fasting insulin (μU/ml)	0.4 ± 0.1	0.7 ± 0.1*
HOMA-IR score	0.7 ± 0.1	1.7 ± 0.3*

*MetS* metabolic syndrome, *LDL* Low-density lipoprotein, *HOMA-IR* Homeostasis model-assessment of insulin resistance

*p ≤ 0.05 vs. Lean

### MSCs-derived EV miRNAs and targeted SA gene regulation in pig models

**Fig. 1:**
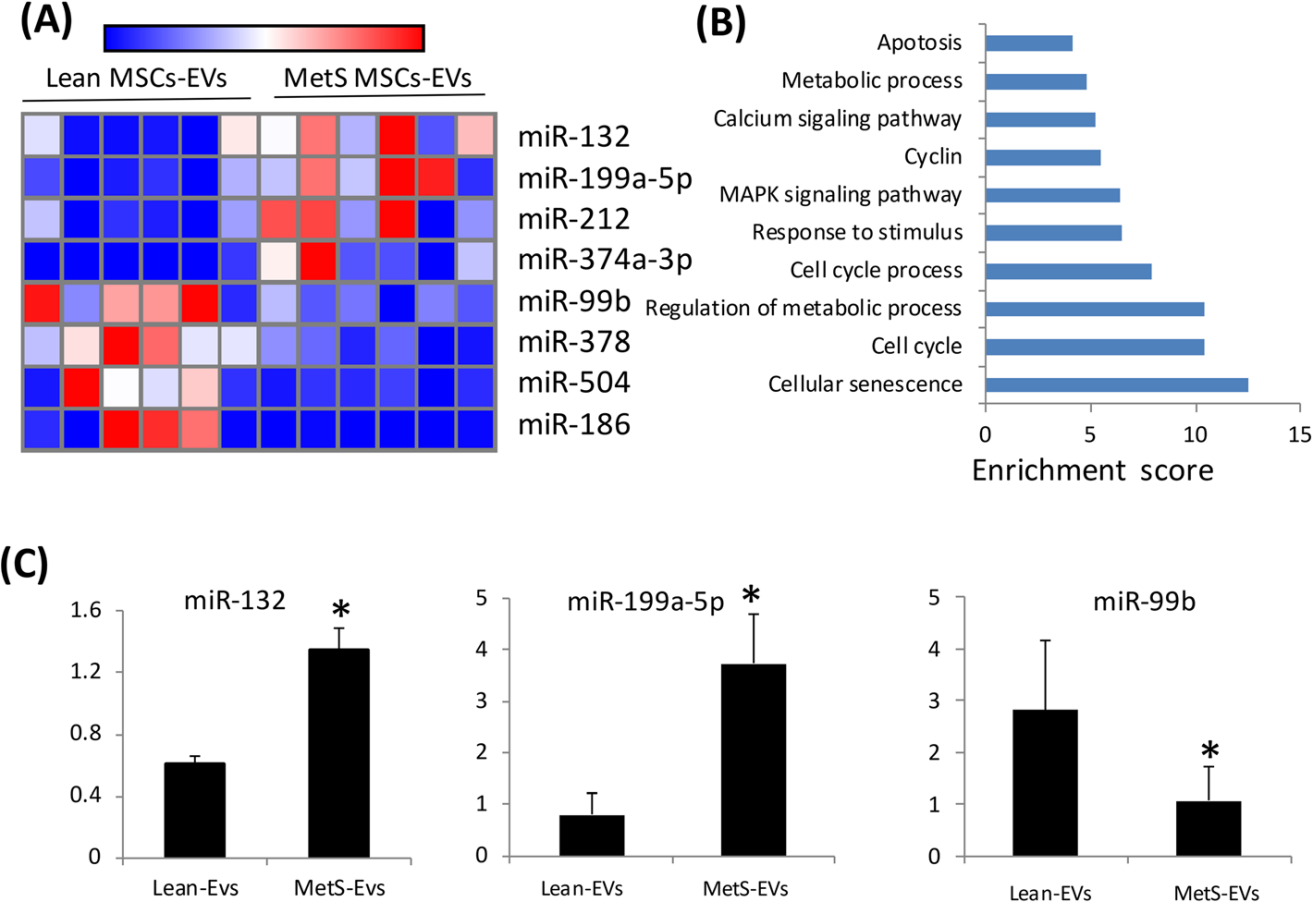
MicroRNA (miRNA) profile in Lean and MetS MSC-EVs in pigs. Heat map showing four upregulated (top) and four downregulated (bottom) miRNAs in MetS compared with Lean MSC-EVs in pigs. Enrichment of functional pathway of the 68 senescence genes targeted by dysregulated pig miRNAs detected using DAVID 6.7. Expression of miR-132, miR-199a-5p, and miR-99b assessed by qPCR was concordant with miRNA-seq findings in pigs

We then analyzed miRNAs in MetS pig MSC-derived EVs. Four miRNAs (miR-132, miR-199a-5p, miR-212, miR-374a-3p) were upregulated and four (miR-99b, miR-378, miR-504, miR-186) downregulated in MetS-MSC-derived EVs compared with Lean MSCs-derived EVs (Fig. 1a). Altogether, these dysregulated miRNAs target 5700 genes, of which 68 are related to cellular senescence. Functional annotation clustering and pathway analysis of these 68 genes, including MAPK1, PTEN, and MTOR, confirmed their relationship with cellular senescence, cell cycle, regulation of metabolic process, and MAPK signaling pathways (Fig. 1b). Expression patterns of miR-199a-5p, miR-132 and miR-99b were subsequently confirmed by PCR (Fig. 1c). The SA-genes targeted by dysregulated miRNAs are detailed in Supplementary Material (Table 1s).

### Systemic characteristics of Lean and Mets patients

Table 2 shows the demographic, clinical, and laboratory characteristics of the study patients. Body mass index, systolic, and diastolic blood pressures were all significantly higher in the MetS than Lean group, whereas age and sex were similar. Total cholesterol, low-density lipoprotein, insulin, and hemoglobin A1C levels were also higher in MetS compared to Lean, underscoring development of MetS. Elevated eGFR was consistent with development of hyperfiltration that characterizes obese individuals.

**Table 2:** Clinical, laboratory, and demographic data of Lean and Mets patients

parameter	Lean	Mets
Number	5	4
Age (years)	24.2 (21–29)	29.3 (24–32)
Gender (female/male)	3/2	2/2
Body mass index	19.1 ± 0.8	60.7 ± 16.2*
SBP (mmHg)	110.6 ± 11.7	147 ± 12.5*
DBP (mmHg)	63.4 ± 5.1	93.8 ± 6.3*
Hemoglobin A1C(%)	5.3 ± 0.2	6.9 ± 1.3*
Total cholesterol (mmol/l)	4.4 ± 0.4	5.3 ± 0.9*
Low-density lipoprotein (mmol/l)	1.6 ± 0.3	3.0 ± 0.4*
Blood urea nitrogen (mmol/l)	3.7 ± 0.4	5.5 ± 1.6*
GFR (ml/min/1.73m^2^)	112.8 ± 34.4	214.5 ± 40.4*
Cystatin-C (mg/1)	0.9 ± 0.1	1.4 ± 0.2*

*SBP* systolic blood pressure, *DBP* diastolic blood pressure, *GFR* estimated glomerular filtration rate

*P < 0.05 vs Lean

### MSCs-derived EVs miRNAs and targeted SA gene regulation in MetS patients

**Fig. 2:**
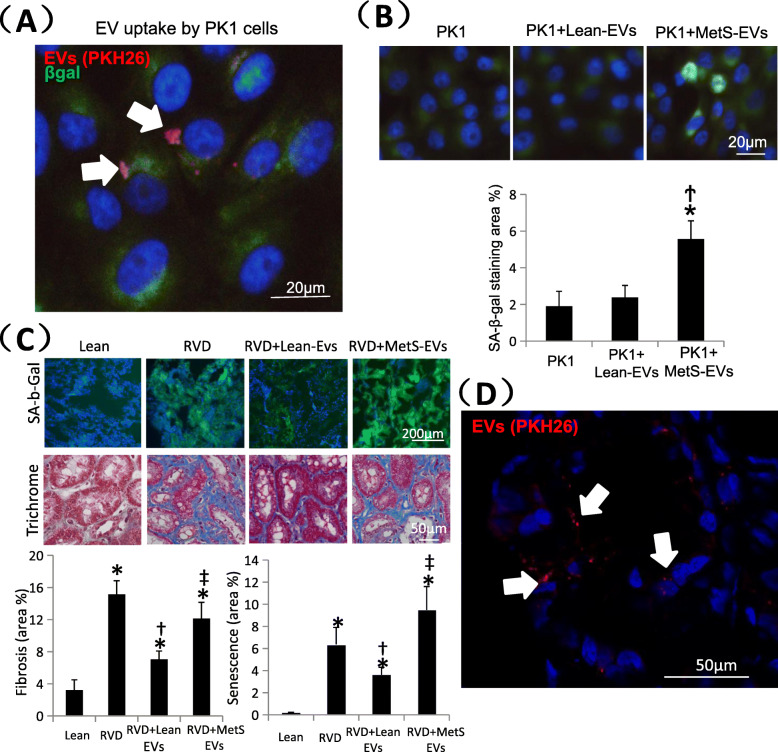
MicroRNA (miRNA) profile in MSC-EVs in human subjects and functional pathway analysis of the common SA-genes. **a** Heat map showed four upregulated (top) and nine downregulated (bottom) miRNAs in MetS compared with Lean MSC-EVs in human subjects. **b** Enrichment of functional pathway of the 131 miRNA-targeted senescence genes using DAVID 6.7 in human. *p < 0.05 vs Lean MSC-EVs. **c** 57 common SA-genes targeted by differentially expressed miRNAs in human and swine MetS-MSCs. **d** Enrichment of functional pathway of the 57 SA-genes using DAVID 6.7

We further analyzed miRNAs dysregulated in human MetS MSC-derived EVs. Four miRNAs (miR-136-3p, miR-4798-5p, miR-12,136, miR-222-3p) were downregulated and nine (miR-630, miR-144-3p, miR-143-5p, miR-4787-3p, miR-769-5p, miR-8074, miR-181a-5p) upregulated in MetS- compared with Lean MSC-derived EVs (Fig. 2a). Altogether, these dysregulated miRNAs target 5700 genes, of which 131 are related to cellular senescence. Functional annotation clustering and pathway analysis of these 131 genes, including PTEN, FOXO3, and MAPK1, showed their relationship with cellular senescence, cell cycle, metabolic processes and apoptosis pathway (Fig. 2b). SA-genes targeted by dysregulated miRNAs in human subjects with MetS are listed in Supplementary Material (Table 2s).

Further comparison of targeted SA-genes of differentially expressed miRNAs in human and swine MetS-MSCs identified 57 common genes, accounting for about 40% of the targeted human SA-genes and 84% of the pig SA-genes (Fig. 2c). Functional annotation clustering and pathway analysis of these 57 common genes disclosed their association with cellular senescence, regulation of metabolic processes, cell cycle, responses to stimulus, apoptosis, and MAPK signaling pathways (Fig. 2d), which are clustered with SA-genes. The common dysregulated miRNAs targeted SA genes are listed in Supplementary Material (Table 3s).

### Systemic characteristics of Lean and RVD pigs

The systemic characteristic of Lean and RVD pigs are summarized in Table 3**.** Treated or untreated RVD pigs developed a similar degree of significant renal artery stenosis and elevated blood pressure. Furthermore, in untreated RVD pigs serum creatinine was significantly elevated compared to Lean pigs.

**Table 3:** Systemic characteristics and single-kidney function in pigs with renovascular disease (RVD) 4 weeks after treatment with MSC-derived extracellular vesicles (EVs)

Parameter	Lean	RVD	RVD+ Lean-EVs	RVD + MetS-EVs
SBP (mmHg)	127.0 ± 15.3	159.8 ± 22.3*	142.3 ± 12.3*	170.8 ± 12.6*
DBP (mmHg)	92.8 ± 11.0	115.0 ± 17.7*	108.0 ± 10.5*	105.7 ± 4.5*
MAP (mmHg)	90.7 ± 4.4	131.9 ± 17.3*	116.8 ± 12.5*	127.4 ± 6.3*
Degree of stenosis (%)	0	64.2 ± 19.6*	65.0 ± 8.4*	65.8 ± 6.6*
Serum creatinine (mg/dL)	1.44 ± 0.21	1.77 ± 0.23*	1.48 ± 0.29†	1.76 ± 0.26*‡

*SBP* systolic blood pressure, *DBP* diastolic blood pressure, *MAP* mean arterial pressure

*n* = 6 each group. *p < 0.05 vs. Lean; *†p* < 0.05 vs. RVD; ‡*p <* 0.05 vs. RVD + Lean-EVs

### MetS attenuates the repair capacity of MSC-derived EVs in vitro and in vivo

**Fig. 3:**
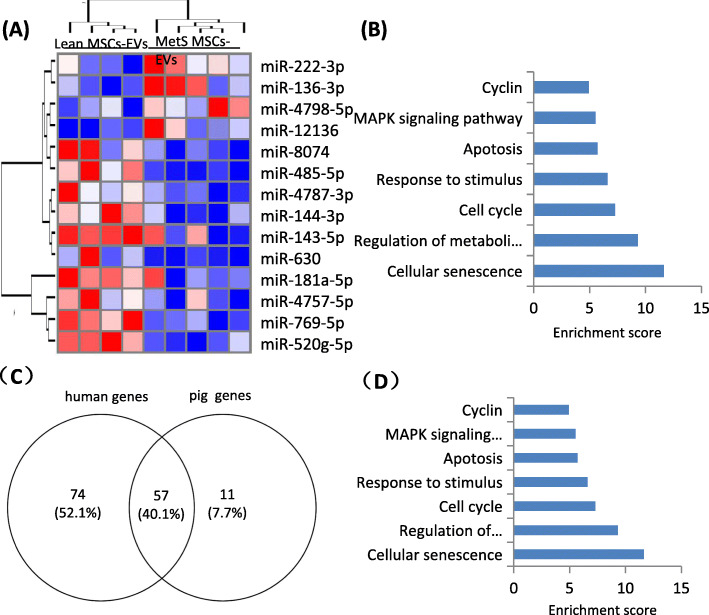
Effects of MSC-derived EVs in PK1 cells and pig kidney. **a** Co-cultured with MetS MSC-EVs, PK1 cells showed higher senescence.*p < 0.05 vs PK1, †*p* < 0.05 vs PK1 + Lean-EVs. **b** PKH-26-labeled EVs (red) were detected in PK1 cells. **c** Representative kidney staining with immunofluorescent SA-b-Gal (left top) and trichrome (left bottom), and respective quantification. Lean EVs attenuated cellular senescence and fibrosis in vivo in injured kidneys, whereas MetS EVs failed to blunt them. **d** Pkh-26-labeled EVs (red) were detected in frozen section in the RVD kidney. *p < 0.05 vs Lean, †*p* < 0.05 vs RVD, ‡*p* < 0.05 vs RVD + Lean-EVs

Labeled EV-rich fractions (red) were identified in PK1 cells (Fig. 3b), confirming their uptake. Senescent cells are characterized by increased enzymatic activities of the lysosomal hydrolase SA-ß-gal. SA-ß-gal staining remained similar to control in PK1 cells co-cultured with Lean-MSC-EVs, but increased significantly in PK1 cells co-cultured with MetS-MSC-EVs (Fig. 3a), indicating that they induced senescence.

RVD kidneys showed elevated SA-ß-gal and trichrome staining (Fig. 3c), indicating development of cellular senescence and fibrosis, as we have shown [7]. In stenotic pig kidneys injected with Lean-MSC-EVs or MetS-MSC-EVs, uptake of labeled EVs (red) was identified (Fig. 3d). Compared with RVD, delivery of Lean-EVs significantly blunted, although not normalized, cellular senescence and fibrosis. On the other hand, delivery of MetS-EVs did not decrease either cellular senescence or fibrosis, which remained not different from the untreated RVD group, and elevated compared with the RVD + Lean-EVs group. Furthermore, Lean-EVs, but not MetS-EVs, also significantly decreased serum creatinine levels (Table 3).

## Discussion

The current study shows that MetS increases the content of senescence-associated miRNAs in MSCs-derived EVs in both a pig model and human subjects. While the specific SA-miRNAs differed between humans and pigs with MetS, their MSCs-derived EVs shared 57 common SA target genes. These observations suggest that increased content of SA-miRNAs in MSCs-derived EVs in MetS is conserved in pigs and humans. Furthermore, we found that pig MetS-EVs induce cellular senescence in PK1 cells, and show dampened potency for attenuation of cellular senescence and fibrosis in RVD pig kidneys. Therefore, the altered SA-content profile of MSCs-derived EVs in MetS may interfere with their ability to blunt senescence in target cells.

MSC-derived EVs mediate the paracrine function of their parental MSCs by transferring physiologically-relevant components from MSCs to recipient cells. The current study extends our previously observation that MetS alters miRNA spectrum in MSCs that might participate in regulation of cellular senescence within the parent MSCs [24], and shows that MetS also modifies miRNA content in their daughter EV paracrine vectors, which might modulate cellular senescence within target cells that MSCs communicate with. We identified miRNAs dysregulated in MetS-MSCs-derived EVs compared with Lean MSC-derived EVs, which target 68 SA-genes. Further functional annotation clustering and pathway analysis showed the relationship of these 68 genes with cellular senescence, cell cycle, regulation of metabolic process, and MAPK signaling pathway, which are also the major senescence pathways targeted by miRNAs in MetS-MSCs in pigs [12]. Hence, miRNAs in EVs might have a similar potential to their parent MSCs in regulating cellular senescence.

This study also underscores that notion that while miRNAs enriched in EVs may be diverse across species, they share a potential to regulate similar pathways in recipient cells, such as proliferation, inflammation, and angiogenesis [25, 26]. While dysregulated miRNAs were not identical in human and pig adipose tissue-derived MSC-EVs, they had comparable targets involved in cellular senescence, cell cycle, metabolic processes, and apoptosis pathways. Remarkably, despite species-dependent heterogeneity, our pig model and patients with MetS shared 57 SA-genes, accounting for 40% of the human targeted SA-genes and 84% of the pig genes, highlighting the similarity of MetS-induced alterations in both species.

A recent study showed that small EVs may contribute to paracrine senescence as key regulators of intercellular communication [27]. EVs play important roles in cell-to-cell communication of their parent cells by transferring miRNAs, DNA, proteins, mRNAs, lipids, and organelles to recipient cells [28]. Among the dysregulated miRNAs, several have been reported to participate in senescence by specific pathways. For example, miR-222-3p can induce cell-cycle phase-arrest and telomere erosion, establishing a senescent phenotype by promoting accumulation of human dermal fibroblasts in G1/S cell cycle phase [29]. On the other hand, miR-143-5p activates AMPK signaling and promotes apoptosis and senescence by targeting the eEF2 gene [30]. Similarly, we found that MetS-EVs could promote cellular senescence in pig PK1 cells in vitro, which might be induced by the dysregulated miRNAs.

The ability of EVs to regulate renal cellular senescence in RVD was unknown. We observed that cellular senescence was increased in untreated RVD kidneys, as we have found before in a different pig model of dyslipidemic RVD [7] and in kidneys of obese mice [31]. The current study demonstrates that EVs derived from healthy (Lean) MSCs attenuate cellular senescence detected by SA-ß-gal in injured post-stenotic swine kidneys. Equivalently, we have recently reported that MSC-derived EVs attenuated cellular senescence and inflammation in the pig myocardium [17]. Finally, similar to cellular senescence, Lean-EVs, but not MetS-EVs, attenuated renal fibrosis. While this does not mandate causality, this observation is consistent with a recent study linking cellular senescence with development of renal fibrosis in tubular epithelial cells [32]. Importantly, decreased tissue injury by Lean-EVs translated into improved kidney function. Overall, our findings suggest that altered content of miRNA targeting SA-genes might be functionally meaningful and restrict their reparative effectiveness.

Our study combined and comprehensively analyzed the data from human and pig models, and has a number of strengths. Using next-generation sequencing analysis, we identified differential miRNA expression signatures in MSC-derived EVs in MetS compared with Lean human subjects and pigs, and subsequently demonstrated their decreased efficacy to attenuate cellular senescence in vivo and in vitro. Our functional analysis focused on dysregulated miRNAs and their targeted SA-genes, and succeeded to demonstrate the role of miRNAS packed in EVs in cellular senescence. The limitations of our study included a relatively small sample size, and short duration of MetS and RVD in pigs, although importantly our patients were also relatively young. In addition, the MSCs and EVs used in this study were harvested in strict adherence with our routine protocols [17], without undergoing further de novo characterization. We also used for RNA-Seq EVs from third passage of MSCs, because at late passages MSCs may become senescent [33]. Further studies are needed to explore in detail genes and molecules that regulate these pathways, as well as techniques to blunt them. The number of delivered EVs (10^11^) resembles the overall concentration of EVs in the systemic circulation (10^9^–10^12^) [34], yet MSC-derived EV are especially endowed with reparative properties and were injected into the renal artery, and thereby achieved momentous effects.

## Conclusions

MetS dysregulates in MSC-derived EVs miRNAs that may participate in regulating cellular senescence in both pigs and human subjects. These alterations result in promotion of senescence by MetS-EVs, and their incompetence to ameliorate senescence and fibrosis in injured kidneys. These studies suggest that miRNAs in MSC-derived EVs may regulate cellular senescence and may have important implications for both the endogenous cellular repair system, as well as for the use of autologous EVs as a therapeutic regenerative strategy.

## Supplementary information

**Additional file 1: Table S1.**

**Additional file 2: Table S2.**

**Additional file 3: Table S3.**

## Data Availability

The authors declare that data supporting the findings of this study are available within the article and its Additional information files.

## Abbreviations

MetS: Metabolic syndrome
MSC: Mesenchymal stem/stromal cells
SA: Senescence-associated
EVs: Extracellular vesicles
RVD: Renovascular disease
miRNA: micro-RNA
SASP: Senescence-associated secretory phenotype
