# Correction to: Metabolic syndrome increases senescence-associated micro-RNAs in extracellular vesicles derived from swine and human mesenchymal stem/stromal cells

**DOI:** 10.1186/s12964-020-00675-x

**Published:** 2020-10-28

**Authors:** Yongxin Li, Yu Meng, Xiangyang Zhu, Ishran M. Saadiq, Kyra L. Jordan, Alfonso Eirin, Lilach O. Lerman

**Affiliations:** 1 Division of Nephrology and Hypertension, Mayo Clinic, 200 First Street SW, Rochester, MN 55905 USA; 2 Department of Vascular Surgery, The Affiliated Hospital of Qingdao University, Qingdao, 266000 People’s Republic of China; 3 Department of Nephrology, The First Hospital Affiliated To Jinan University, Guangzhou, 510630 People’s Republic of China

## Correction to: Cell Commun Signal 18:124 (2020) 10.1186/s12964-020-00624-8

Following publication of the original article [1], the authors identified an error in Figs. 2 and 3. The two figures have been mistakenly transposed. The correct Figs. 2 and 3 have been presented below:

**Fig. 2:**
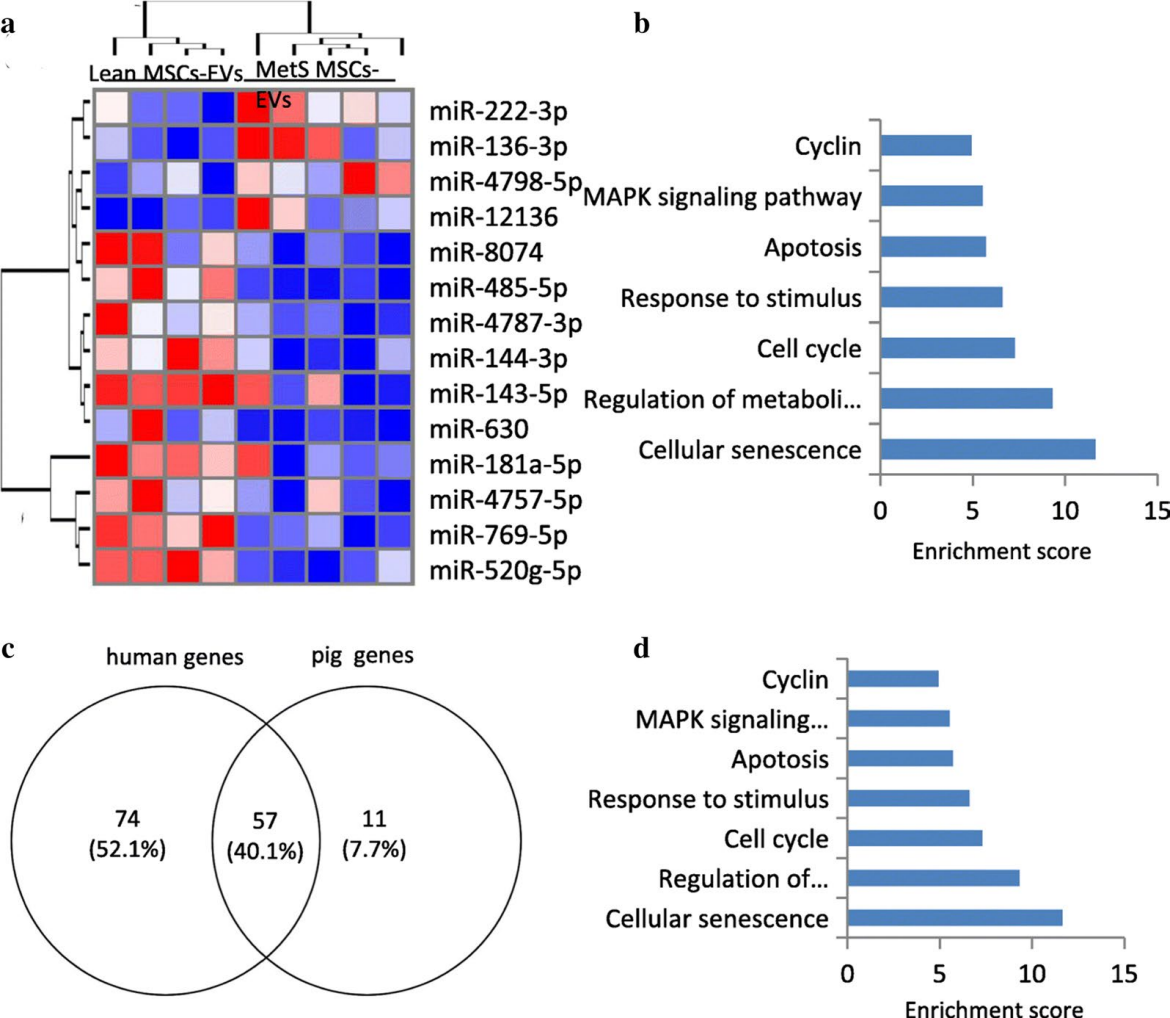
MicroRNA (miRNA) profile in MSC-EVs in human subjects and functional pathway analysis of the common SA-genes. **a** Heat map showed four upregulated (top) and nine downregulated (bottom) miRNAs in MetS compared with Lean MSC-EVs in human subjects. **b** Enrichment of functional pathway of the 131 miRNA-targeted senescence genes using DAVID 6.7 in human. *p < 0.05 vsersu Lean MSC-EVs. **c** 57 common SA-genes targeted by differentially expressed miRNAs in human and swine MetS-MSCs. **d** Enrichment of functional pathway of the 57 SA-genes using DAVID 6.7

**Fig. 3:**
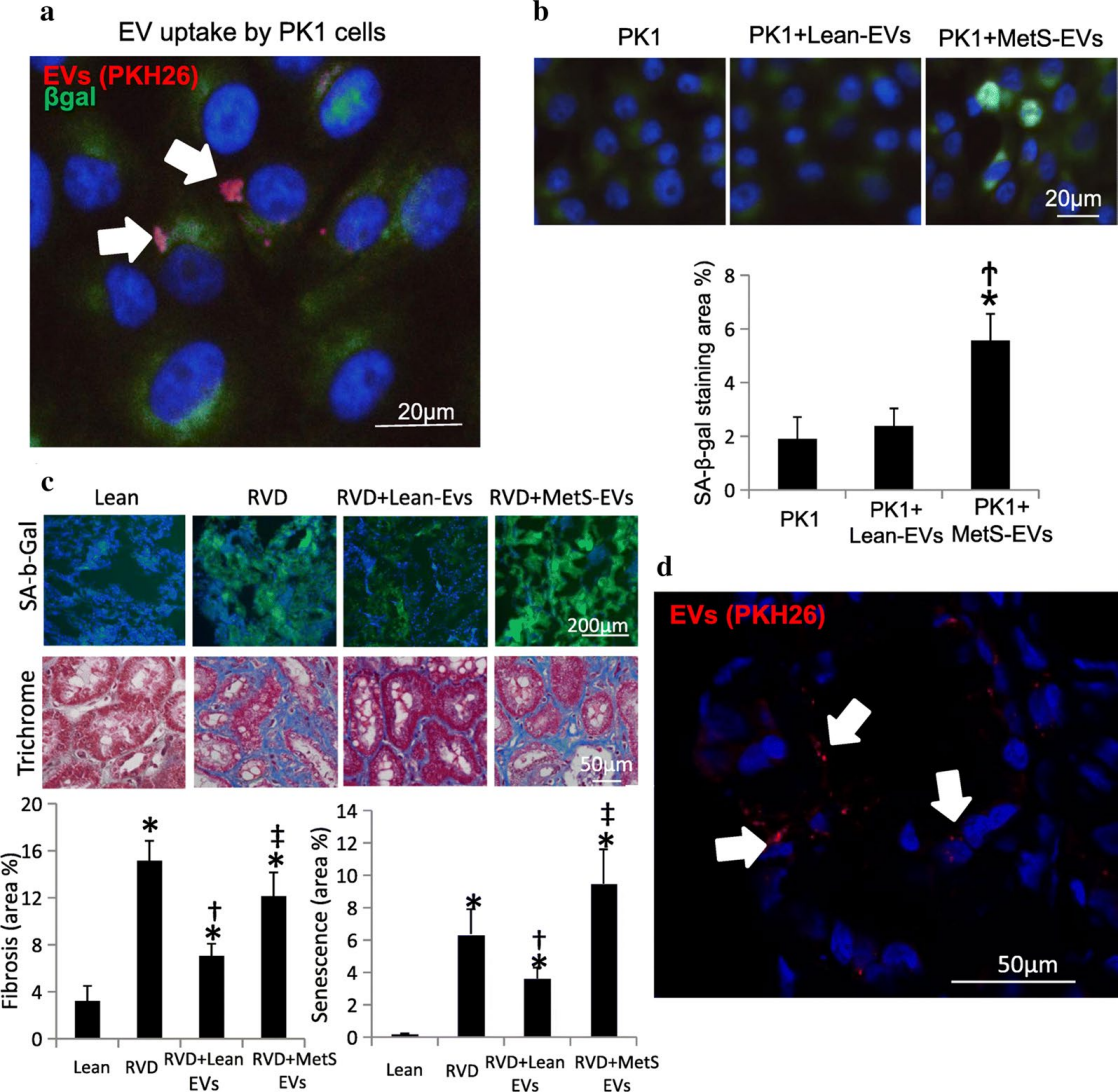
Effects of MSC-derived EVs in PK1 cells and pig kidney. **a** Co-cultured with MetS MSC-EVs, PK1 cells showed higher senescence.*p < 0.05 vs PK1, ^†^*p* < 0.05 vs PK1 + Lean-EVs. **b** PKH-26-labeled EVs (red) were detected in PK1 cells. **c** Representative kidney staining with immunofluorescent SA-b-Gal (left top) and trichrome (left bottom), and respective quantification. Lean EVs attenuated cellular senescence and fibrosis in vivo in injured kidneys, whereas MetS EVs failed to blunt them. **d** Pkh-26-labeled EVs (red) were detected in frozen section in the RVD kidney. *p < 0.05 versus Lean, ^†^*p* < 0.05 vsersu RVD, ^‡^*p*< 0.05 versus RVD + Lean-EVs
