# Supplementary material for: Metabolic syndrome increases senescence-associated micro-RNAs in extracellular vesicles derived from swine and human mesenchymal stem/stromal cells
Source: Cell Commun Signal. 2020 Aug 12;18:124. doi: 10.1186/s12964-020-00624-8 (PMC7425605; doi:10.1186/s12964-020-00624-8)
Supplement: Supplementary file 2 — Additional file 1: Table S1. [file 12964_2020_624_MOESM2_ESM.docx]

**Table 1s**. List of the 68 senescence-associated genes targeted by dysregulated miRNAs in MetS pigs MSC-derived EVs.

| AKT3 | ETS1 | MDM2 | RB1 |
| --- | --- | --- | --- |
| ATM | FBXW11 | MTOR | RBBP4 |
| BTRC | FOXM1 | NFATC2 | RBL1 |
| CALM1 | FOXO1 | NRAS | RHEB |
| CALM3 | FOXO3 | PIK3CA | SERPINE1 |
| CALML4 | HIPK3 | PIK3CB | SIRT1 |
| CAPN2 | HLA-A | PIK3R1 | SLC25A5 |
| CCNB3 | HUS1 | PIK3R3 | SMAD2 |
| CCND1 | IGFBP3 | PPP1CB | TGFB1 |
| CCND2 | ITPR2 | PPP1CC | TGFB2 |
| CCND3 | KRAS | PPP3CA | TGFBR1 |
| CCNE2 | LIN52 | PPP3CB | TGFBR2 |
| CDK6 | LIN54 | PPP3CC | TP53 |
| CDKN1A | MAP2K6 | PPP3R2 | TRPM7 |
| CHEK1 | MAPK1 | PTEN | TSC1 |
| E2F3 | MAPK14 | RAD1 | ZFP36L1 |
| E2F5 | MAPK3 | RAD50 | ZFP36L2 |
