# Supplementary material for: Metabolic syndrome increases senescence-associated micro-RNAs in extracellular vesicles derived from swine and human mesenchymal stem/stromal cells
Source: Cell Commun Signal. 2020 Aug 12;18:124. doi: 10.1186/s12964-020-00624-8 (PMC7425605; doi:10.1186/s12964-020-00624-8)
Supplement: Supplementary file 3 — Additional file 2: Table S2. [file 12964_2020_624_MOESM3_ESM.docx]

**Table 2s.** List of the 131 senescence-associated genes targeted by dysregulated miRNAs MetS patients MSC-derived EVs.

| AKT1 | CDK4 | GATA5 | LIN9 | NFATC4 | RAF1 | TP53 |
| --- | --- | --- | --- | --- | --- | --- |
| AKT2 | CDK6 | HIPK1 | MAP2K1 | NRAS | RBBP4 | TRAF3IP2 |
| AKT3 | CDK7 | HIPK2 | MAP2K2 | PIK3CA | RBBP5 | TRAF3IP3 |
| ATM | CDKN1A | HIPK3 | MAP2K3 | PIK3CB | RBL1 | TRPM7 |
| BTRC | CDKN2B | HIPK4 | MAP2K4 | PIK3CD | RBL2 | TRPM8 |
| CACNA1D | CHEK1 | HIPK5 | MAP2K6 | PIK3R1 | RELA | TRPV4 |
| CALM1 | E2F1 | HLA-A | MAP2K7 | PIK3R2 | RRAS2 | TSC1 |
| CALM2 | E2F2 | HLA-E | MAPK1 | PIK3R3 | SERPINE1 | TSC2 |
| CALM3 | E2F3 | HLA-F | MAPK11 | PPID | SIRT1 | ZFP36L1 |
| CALML4 | E2F4 | HUS1 | MAPK12 | PPP1CB | SLC25A4 | ZFP36L2 |
| CALML5 | E2F5 | IGFBP3 | MAPK13 | PPP1CC | SLC25A6 | ZFP36L3 |
| CAPN1 | EIF4EBP1 | IL1A | MAPK14 | PPP3CB | SMAD2 |  |
| CCNA2 | EIF4EBP2 | ITPR1 | MAPK2 | PPP3R1 | SMAD3 |  |
| CCNB1 | ETS1 | ITPR2 | MCU | PPP3R2 | SMAD4 |  |
| CCND1 | FBXW11 | KRAS | MDM2 | PTEN | SQSTM1 |  |
| CCND2 | FOXO1 | LIN10 | MDM3 | RAD1 | TGFB2 |  |
| CCND3 | FOXO3 | LIN52 | MRAS | RAD50 | TGFB3 |  |
| CCNE2 | FOXO4 | LIN53 | NBN | RAD51 | TGFB4 |  |
| CDK1 | GADD45G | LIN54 | NFATC2 | RAD9A | TGFBR1 |  |
| CDK2 | GATA4 | LIN55 | NFATC3 | RAD9B | TGFBR2 |  |
