# Supplementary material for: Metabolic syndrome increases senescence-associated micro-RNAs in extracellular vesicles derived from swine and human mesenchymal stem/stromal cells
Source: Cell Commun Signal. 2020 Aug 12;18:124. doi: 10.1186/s12964-020-00624-8 (PMC7425605; doi:10.1186/s12964-020-00624-8)
Supplement: Supplementary file 4 — Additional file 3: Table S3. [file 12964_2020_624_MOESM4_ESM.docx]

**Table 3s.** List of the 68 common senescence-associated genes that are targeted by miRNAs found to be dysregulated in MSC-derived EVs harvested from both MetS pigs and patients.

| AKT3 | CDK6 | HLA-A | MDM2 | PPP3R2 | TGFBR1 |
| --- | --- | --- | --- | --- | --- |
| ATM | CDKN1A | HUS1 | NFATC2 | PTEN | TGFBR2 |
| BTRC | CHEK1 | IGFBP3 | NRAS | RAD1 | TP53 |
| CALM1 | E2F3 | ITPR2 | PIK3CA | RAD50 | TRPM7 |
| CALM3 | E2F5 | KRAS | PIK3CB | RBBP4 | TSC1 |
| CALML4 | ETS1 | LIN52 | PIK3R1 | RBL1 | ZFP36L1 |
| CCND1 | FBXW11 | LIN54 | PIK3R3 | SERPINE1 | ZFP36L2 |
| CCND2 | FOXO1 | MAP2K6 | PPP1CB | SIRT1 |  |
| CCND3 | FOXO3 | MAPK1 | PPP1CC | SMAD2 |  |
| CCNE2 | HIPK3 | MAPK14 | PPP3CB | TGFB2 |  |
